# Spontaneous Isthmic Heterotopic Pregnancy Presenting as Suspected Appendicitis With Successful Term Delivery: A Case Report

**DOI:** 10.7759/cureus.105899

**Published:** 2026-03-26

**Authors:** Vithura Kunarathnam, Quyen Kelly, Ananias Motta-Burgos, Merrai Asad, Itai Itzhak, Petr Itzhak

**Affiliations:** 1 Obstetrics and Gynecology, Nassau University Medical Center, East Meadow, USA; 2 Medical School, American University of the Caribbean School of Medicine, New York, USA; 3 Obstetrics and Gynecology, St. John's University, New York, USA

**Keywords:** appendicitis, case report, diagnostic laparoscopy, ectopic pregnancy, first trimester pregnancy, heterotopic pregnancy, intrauterine pregnancy, isthmic pregnancy, spontaneous conception, tubal ectopic pregnancy

## Abstract

Heterotopic pregnancy (HP) is a rare but potentially life-threatening condition defined by the simultaneous presence of intrauterine and extrauterine pregnancies. Spontaneous HPs without identifiable risk factors are particularly uncommon and pose significant diagnostic challenges for clinicians. We report a rare case of a spontaneously conceived isthmic HP in a patient at approximately 11 weeks of gestation who presented with acute abdominal pain. The patient had a confirmed live intrauterine pregnancy (IUP) and no identifiable risk factors for ectopic pregnancy. She was initially evaluated for suspected acute appendicitis and subsequently underwent emergent diagnostic laparoscopy. Intraoperatively, a bleeding isthmic ectopic pregnancy was identified, and a right salpingectomy was performed. The IUP remained viable following surgical management, and the patient ultimately delivered a healthy neonate at 39 weeks’ gestation without complications. This case highlights several unusual features, including spontaneous conception in a patient without identifiable risk factors, diagnosis of an isthmic HP at nearly 11 weeks of gestation, and successful term delivery of the IUP following surgical management. It also emphasizes the importance of maintaining clinical suspicion for HP in patients presenting with abdominal pain during early pregnancy, even when a live IUP has already been confirmed.

## Introduction

Heterotopic pregnancy (HP) is defined as the simultaneous presence of intrauterine and extrauterine pregnancies. Although rare, it represents a potentially life-threatening condition that requires prompt recognition. The estimated incidence is approximately one in 30,000 in spontaneously conceived pregnancies and up to one in 100 in pregnancies conceived through assisted reproductive technologies (ART) [1-4]. The most common site of ectopic implantation is the ampullary portion of the fallopian tube, followed by the cornual region. In contrast, implantation within the isthmic portion is uncommon, accounting for approximately 6% of cases. Although up to 70% of cases result in a term intrauterine delivery, delayed diagnosis and management can significantly increase maternal and fetal morbidity and mortality [5].

Many patients with HP are asymptomatic, and cases are frequently identified during early ultrasound evaluation in patients undergoing ART. When symptoms occur, approximately 70% of patients present with abdominal pain and 50% present with vaginal bleeding [6]. Most patients with HP have identifiable risk factors related to tubal pathology, including a history of sexually transmitted infections, pelvic inflammatory disease, previous ectopic pregnancy, prior abdominal or tubal surgery, or infertility treatments. It has been estimated that at least 71% of patients have at least one of these risk factors.

Consequently, spontaneous HP in patients without identifiable risk factors remains exceedingly rare and may lead clinicians to underestimate the diagnosis once a viable intrauterine pregnancy (IUP) is identified [6]. Among previously reported cases of spontaneous HP without identifiable risk factors, most are diagnosed before eight weeks of gestation, making later presentation particularly uncommon [6,7]. Importantly, the presence of a confirmed IUP may lead clinicians to prematurely exclude ectopic pregnancy, contributing to delayed diagnosis.

The diagnosis of HP is often delayed once a live IUP has been confirmed on ultrasound, as clinicians may prematurely exclude the possibility of a concurrent ectopic pregnancy. Differential diagnoses for pelvic pain in pregnant patients include acute appendicitis, ovarian torsion, and hemorrhagic ovarian cysts. Acute appendicitis remains the most common non-obstetric surgical emergency during pregnancy [8]. Transvaginal ultrasound is the recommended initial imaging modality due to its accessibility and safety profile in pregnancy; however, its sensitivity may be limited, particularly in later gestational presentations or when adnexal findings are subtle. Magnetic resonance imaging (MRI) may serve as a useful adjunct in hemodynamically stable patients when ultrasound findings are inconclusive.

Management of HP requires balancing the treatment of the ectopic pregnancy while preserving the viability of the IUP. Methotrexate is contraindicated in patients with a viable IUP. In select cases, conservative or expectant management may be considered when the patient is asymptomatic, the ectopic pregnancy is unruptured, and beta-human chorionic gonadotropin (β-hCG) levels are decreasing or plateauing. One reported case described successful conservative management in a mildly symptomatic patient who was discharged 20 days later with a viable IUP and a partially resorbed ectopic pregnancy [9]. Surgical management options include ultrasound-guided selective embryo reduction via embryo aspiration, potassium chloride injection, salpingostomy, and salpingectomy [10]. Although a definitive gold standard surgical approach has not been established, operative management remains the preferred treatment in patients with suspected rupture. Patients must be counseled regarding the risk of disrupting the IUP. The reported rate of viable intrauterine delivery following surgical management of HP is approximately 66% [11]. Reported outcomes across studies demonstrate viable IUP rates ranging from approximately 60-70%, highlighting that timely intervention can preserve fetal viability in a significant proportion of cases.

## Case presentation

We present a 26-year-old Hispanic female, gravida 4 para 1021, at 10 weeks and six days of gestation (estimated delivery date (EDD) 07/25/2024 by last menstrual period (LMP) consistent with first-trimester ultrasound), who presented to the Emergency Department (ED) with acute abdominal pain. She reported one day of sharp, diffuse pelvic pain rated 8/10, unrelieved by acetaminophen, along with vaginal spotting (two pads over 24 hours, without passage of clots or tissue). Associated symptoms included dysuria for one week and subjective fever for one day. She also reported decreased appetite without nausea or vomiting.

Her obstetric history was notable for one prior term vaginal delivery (2019), one elective termination (2022), and one spontaneous abortion (2023). This pregnancy was conceived spontaneously. She denied prior sexually transmitted infections and had no significant past medical history. Surgical history included suction, dilation, and curettage in 2022. She denied tobacco use.

**Figure 1:**
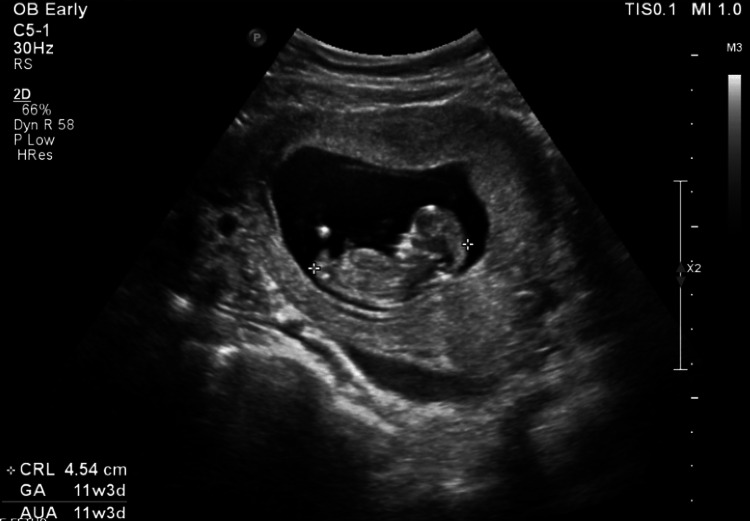
Transabdominal ultrasound demonstrating a live intrauterine pregnancy with fetal cardiac activity.

**Figure 2:**
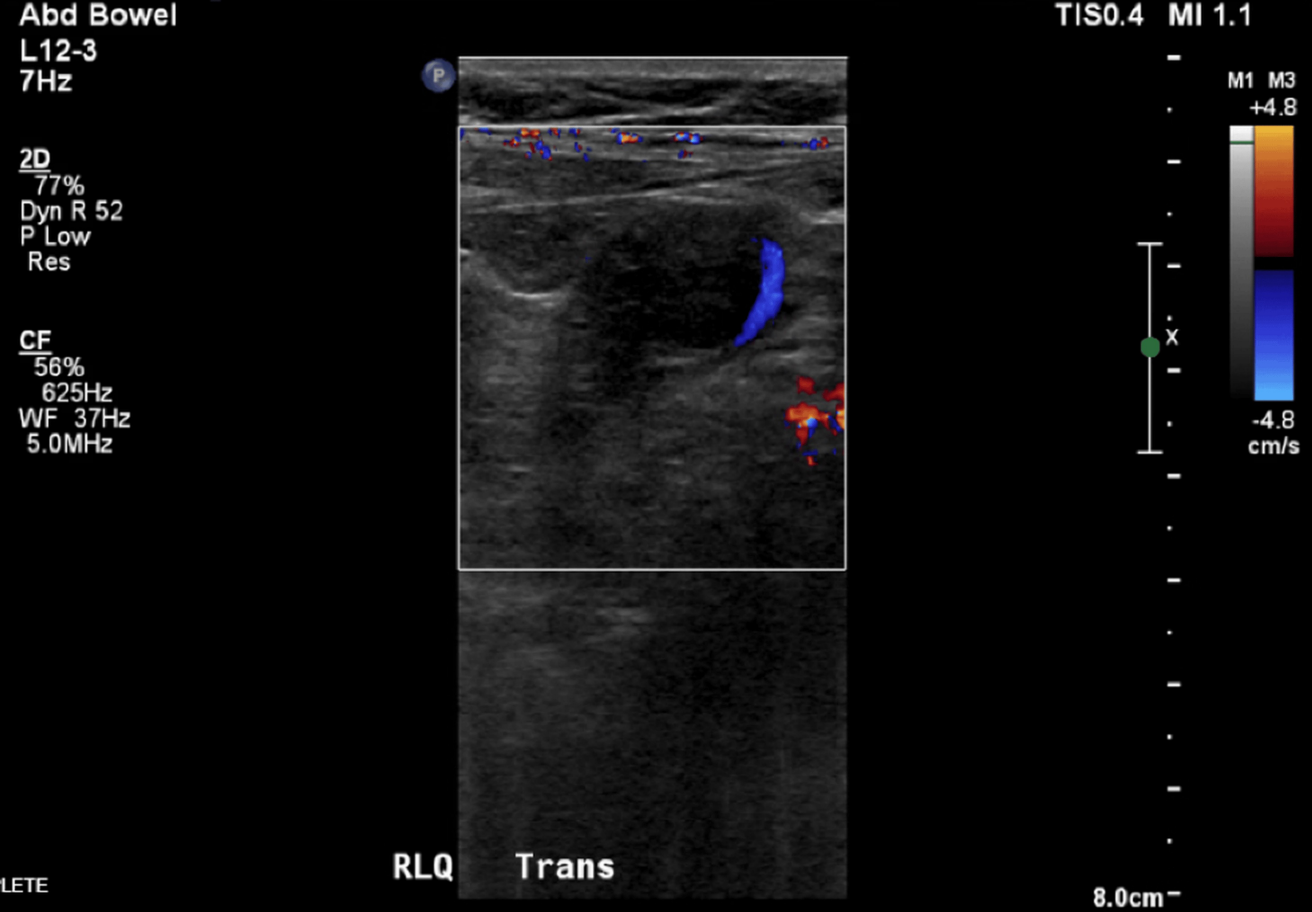
Ultrasound image showing a tubular hollow viscus structure in the right lower quadrant, raising concern for appendiceal or bowel pathology.

Pelvic ultrasound demonstrated a single live IUP at 11 weeks and three days with a fetal heart rate of 132 beats per minute (Figure 1). A small subchorionic hematoma and trace pelvic free fluid were noted. Both ovaries appeared normal with preserved Doppler flow. A tubular structure was visualized in the right lower quadrant; however, the appendix could not be clearly identified (Figure 2). The patient declined a transvaginal ultrasound.

On examination, the patient appeared in acute distress. The abdomen was soft with diffuse tenderness and mild guarding, without rebound tenderness. Speculum examination revealed minimal blood in the vaginal vault with a closed cervix. Laboratory results were unremarkable, including a normal white blood cell count. General surgery was consulted for suspected appendicitis, and an MRI was planned.

Within one hour, the patient developed worsening abdominal pain with increased guarding, rebound tenderness, and marked right lower quadrant tenderness. Psoas and obturator signs were positive. Given concern for acute abdomen, a multidisciplinary decision was made to proceed with emergent surgical exploration.

**Figure 3:**
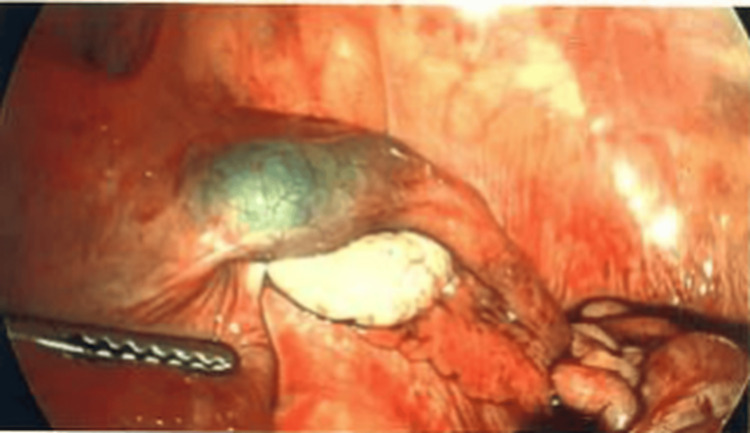
Laparoscopic visualization of the right fallopian tube demonstrating a dilated isthmic segment with suspected ectopic pregnancy.

Diagnostic laparoscopy revealed a normal-appearing uterus, left fallopian tube, and bilateral ovaries. The appendix appeared normal. The right fallopian tube was dilated at the isthmic portion with active bleeding (Figure 3), and approximately 200 mL of hemoperitoneum was evacuated. Due to ongoing bleeding, a right salpingectomy was performed. The patient remained hemodynamically stable postoperatively, and fetal Doppler confirmed a viable IUP. She was discharged with routine follow-up.

**Figure 4:**
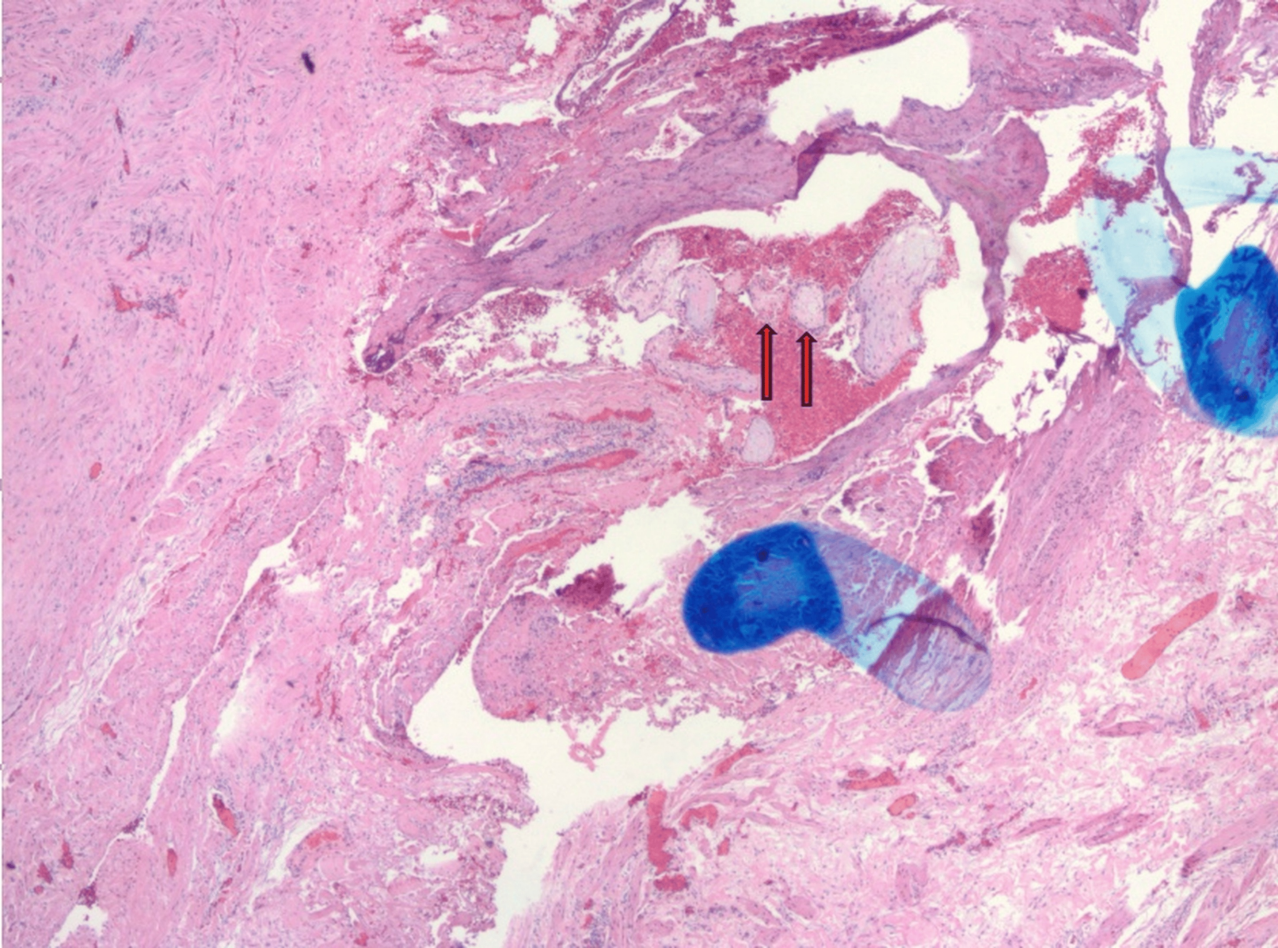
Histopathologic image of the excised fallopian tube demonstrating immature chorionic villi (indicated by arrows) embedded within the tubal wall, consistent with ectopic pregnancy.

Histopathologic examination of the excised fallopian tube demonstrated immature chorionic villi (Figure 4), confirming ectopic pregnancy.

## Discussion

The diagnosis of HP remains one of the greatest challenges in gynecologic emergencies. It is often delayed due to the early visualization of an intrauterine sac, which can create a false sense of reassurance for clinicians [12]. The initial presentation may be confused with other non-gynecologic pathologies due to overlapping symptomatology. Additionally, clinical symptoms may be masked by a concurrent spontaneous abortion of the IUP. Importantly, confirmation of a viable IUP on ultrasound should not exclude the possibility of a concurrent ectopic pregnancy, particularly in patients presenting with persistent abdominal pain.

An HP located in the isthmic portion of the fallopian tube typically remains undetected until rupture occurs. In this muscular portion of the tube, the wall has greater compliance and can withstand dilation to accommodate a larger gestational age. However, as the ectopic pregnancy enlarges, increasing vascularity may result in catastrophic maternal hemorrhage [11]. Similar cases of HP presenting with symptoms concerning for acute appendicitis have also been reported in the literature, further highlighting the diagnostic challenge of this condition [13].

To our knowledge, only a limited number of cases of spontaneously conceived isthmic HP diagnosed beyond eight weeks of gestation have been reported in the literature, making this presentation particularly uncommon. This case is notable for several reasons. First, the HP occurred in the isthmic portion of the fallopian tube, an uncommon location representing approximately 6% of tubal ectopic pregnancies. Second, the pregnancy was conceived spontaneously in a patient without identifiable risk factors for ectopic pregnancy. Third, the diagnosis occurred at nearly 11 weeks of gestation, which is later than most reported cases of spontaneous HP in the literature. Finally, despite the need for emergent surgical management due to active bleeding and hemoperitoneum, the IUP remained viable and ultimately resulted in an uncomplicated term delivery.

In our case, the patient had presented to the ED multiple times with similar complaints. Her initial visit occurred at five weeks of gestation with abdominal pain and vaginal bleeding. An ultrasound at that time demonstrated a live IUP with a subchorionic hematoma. Her symptoms were mild, and she was diagnosed with a threatened abortion and discharged with return precautions. Approximately one month later, she returned to the ED with complaints of nausea, vomiting, and mild pelvic pain. A pelvic ultrasound again demonstrated a live IUP with an unchanged subchorionic hematoma and no additional abnormalities. Her pelvic pain during this visit was attributed to dehydration and muscular contractions related to vomiting. The patient exhibited mild tenderness on abdominopelvic examination during both visits. In retrospect, these recurrent ED visits highlight the importance of maintaining a broad differential diagnosis when symptoms persist in early pregnancy, even when prior imaging has demonstrated a viable IUP.

This case also underscores an important clinical consideration in the evaluation of early pregnancy. Although visualization of a viable IUP is often reassuring, it should not lead clinicians to prematurely exclude the possibility of a concurrent ectopic pregnancy. Persistent or worsening abdominal pain in early pregnancy should prompt careful evaluation of the adnexa and consideration of HP, even in patients without traditional risk factors. This case provides a practical clinical framework for recognizing atypical presentations of HP in the emergency setting.

Transvaginal ultrasound is the recommended initial imaging modality because it allows evaluation of both the intrauterine cavity and bilateral adnexa. MRI may be considered in hemodynamically stable patients when further evaluation of abdominal pathology is required. However, in patients with worsening symptoms or signs of an acute abdomen, prompt surgical exploration remains critical to prevent maternal morbidity from rupture and hemorrhage. The goals of treatment include early detection and intervention of the ectopic pregnancy while minimizing disruption of the IUP, preserving fertility, and reducing the risk of recurrence. Management of these cases remains complex, but each component is essential in reducing maternal morbidity and mortality while maximizing the likelihood of a successful IUP outcome.

In this case, the HP was identified only after the patient’s clinical condition deteriorated, requiring emergent surgical intervention. Histopathologic confirmation with identification of chorionic villi within the fallopian tube remains the gold standard for diagnosis and is essential in confirming ectopic implantation. The pregnancy was subsequently followed with routine prenatal care, and the patient ultimately delivered a healthy full-term neonate at 39 weeks of gestation without complications. This outcome demonstrates that timely surgical management of the ectopic component of HP can result in favorable maternal and fetal outcomes.

A review of previously published case reports demonstrates that spontaneous HP remains rare, particularly in patients without identifiable risk factors, and most cases are diagnosed during the early first trimester (Table 1).

**Table 1:** Selected reports of spontaneous heterotopic pregnancy from the literature, highlighting the rarity of late diagnosis and isthmic implantation in spontaneously conceived pregnancies. IUP: intrauterine pregnancy

Author	Year	Gestational Age at Diagnosis	Location	Management	Outcome of IUP
Tandon et al. [14]	2009	6-7 weeks	Tubal	Salpingectomy	Viable IUP
Černiauskaitė et al. [6]	2020	7 weeks	Tubal	Laparoscopic salpingotomy	Term delivery
Lamrissi et al. (Case series; total of 5 cases) [12]	2022	Varied (mean ~8 weeks)	Tubal	Surgical management	Variable outcomes
Valencia et al. [15]	2024	7 weeks	Tubal	Salpingectomy	Viable IUP
Wang et al. [16]	2025	12 weeks	Tubal	Salpingectomy	Viable IUP
Present case	2024	10-11 weeks	Isthmic	Salpingectomy	Term delivery at 39 weeks

## Conclusions

This case highlights the diagnostic challenges of HP in patients with a confirmed IUP. Our patient had no identifiable risk factors on history, physical examination, or imaging that suggested HP as a primary diagnostic consideration. Clinicians should maintain a high index of suspicion for HP in reproductive-age patients presenting with abdominal pain or vaginal bleeding, particularly those with recurrent ED visits or persistent symptoms despite prior reassuring imaging. The presence of an IUP on ultrasound does not exclude the coexistence of an ectopic pregnancy. Early recognition and timely intervention are essential to reduce maternal morbidity and improve the likelihood of a favorable obstetrical outcome. This case further highlights that early recognition and appropriate management of HP can result in favorable maternal and fetal outcomes, even in rare and diagnostically challenging presentations.
